# Select Configurational Dynamics in Ethanolamine Ammonia-Lyase Radical Enzyme Catalysis

**DOI:** 10.1021/acsphyschemau.5c00051

**Published:** 2025-09-02

**Authors:** Wei Li, Andrew M. Stewart, Kurt Warncke

**Affiliations:** Department of Physics, 1371 Emory University, Atlanta, Georgia 30322, United States

**Keywords:** enzyme catalysis, protein dynamics, solvent dynamics, molecular mechanism, electron paramagnetic resonance

## Abstract

Contributions of protein and coupled solvent configurational fluctuations to the molecular mechanism of enzyme catalysis are addressed in the adenosylcobalamin (coenzyme B_12_)-dependent ethanolamine ammonia-lyase (EAL) enzyme from *Salmonella enterica* serovar Typhimurium. Full-spectrum, time-resolved electron paramagnetic resonance (EPR) spectroscopy is used to measure the temperature dependence of the first-order kinetics of the substrate radical reaction in EAL surrounded by successive hydration and aqueous-cosolvent (aminoethanol; added dimethyl sulfoxide, glycerol, and sucrose) layers in frozen solution. At different temperature (*T*) values in each system, the piecewise-continuous Arrhenius relation displays the characteristic bifurcation, from high-*T* monoexponential dependence (reaction from substrate radical macrostate, **S** ^**•**^) to the low-*T* biexponential dependence (reaction from sequential substates, **S**_**1**_^**•**^ and **S**_**2**_^**•**^). Parallel measurements of the solvent dynamics around EAL by using EPR spin probe mobility or electric permittivity detect a dynamical transition from collective cluster to individual fluctuations of coupled protein surface groups and hydration water, with decreasing *T*, that precisely coincides with the kinetic bifurcation *T* in each solvent system. When shifted along their common monotonic high-*T* relation, the Arrhenius dependences collapse onto a single, universal pattern. The results indicate that specific, or select, collective fluctuations in the EAL protein hydration layer are coupled to active-site configurational fluctuations, providing low-barrier portals through the configuration space. The direct mechanistic link between solvent dynamics and turnover expands the understanding of radical-mediated reactions in EAL and supports the model that select collective configurational dynamics are a fundamental feature of enzyme catalysis.

## Graphical abstract

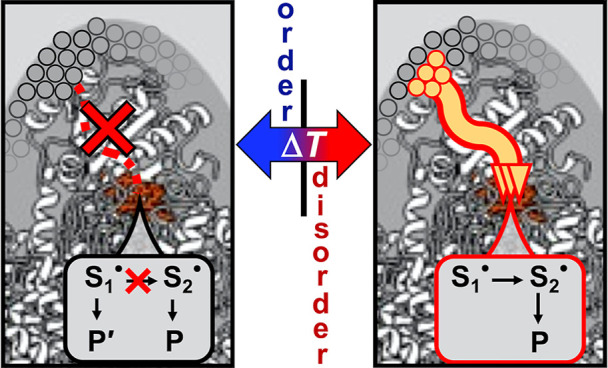

## Introduction

The molecular mechanism of the core chemical reaction sequence in enzymes involves a hierarchy of configurational states,^1^ in common with other protein functions.^2,3^ At the top, a sequence of canonical macrostates corresponds to free energy minima separated by relatively large barriers, one of which generally defines the rate-limiting step in the catalytic cycle. Macrostates are comprised of substates that are separated by lower energy barriers. Substates may display structural features involved in the conduct of preceding and subsequent steps.^4,5^ On a finer level, configurational microstates provide the structural continuum between substates.^5–7^ The thermally driven, stochastic configurational fluctuations among the microstates that span and link substates occur on time scales several orders of magnitude shorter than the characteristic times of the chemical reaction steps between macrostates and, thus, are not detected directly by ensemble-based, steady-state, or transient kinetics measurements at physiological temperature (*T*) values.^8,9^ Here, we use controlled confinement at low *T* and varied aqueous solvent composition to resolve and characterize the mechanism and specific contributions of the microstate configurational dynamics to the enzyme catalytic sequence, by using the adenosylcobalamin (coenzyme B_12_)-dependent ethanolamine ammonia-lyase (EAL).

EAL [EC 4.3.1.7; cobalamin (vitamin B_12_)-dependent enzyme superfamily] catalyzes the conversion of aminoethanol to acetaldehyde and ammonia by using a radical mechanism (Figure 1).^10,11^ EAL is the lead enzyme in the bacterial ethanolamine utilization (Eut) metabolic pathway, which is associated with microbiome homeostasis and disease conditions in the human gut.^12–14^ The core chemical bond-making/bond-breaking transformations of substrate to product in EAL involve an astoundingly versatile sequence, initiated by rate-limiting C–N bond scission^15^ in the substrate radical, followed by 1,2-sigmatropic migration of the amino group to form the product radical, dissociation of the amino group to form the acetaldehyde radical and ammonia, and quenching of the radical by return hydrogen atom transfer from 5′-deoxyadenosine (Ad-CH_3_), which leads to diamagnetic product. We have measured the temperature step-initiated, first-order reaction kinetics of the cryotrapped aminoethanol substrate radical in a low-*T* system (Figure 1), which affords *T* variation over an exceptional range of 190–230 K, extended to 295 K by using measured *k*_cat_ values corresponding to the same rate-limiting step.^17–19^ The substrate radical macrostate (**S** ^**•**^) is resolved by a bifurcation of the linear low-*T* Arrhenius dependence of the decay rate constant into two sequential substates:^18,19^ (1) The capture substate (**S**_**1**_^**•**^) stabilizes the initial substrate radical species formed by hydrogen abstraction by the 5′-deoxyadenosyl radical. (2) The enabled substate (**S**_**2**_^**•**^) is set to actuate the reaction to form the product radical. The pathway of configurational microstates through the **S**_**1**_^**•**^ and **S**_**2**_^**•**^ substates was tracked by measuring the reactivity of the microstates over a 10^4^-fold range of first-order rate constant.^20^ This reactivity tracking showed that **S**_**1**_^**•**^ is an ensemble, composed of a large number of microstates, consistent with the role of configurational entropy in stabilizing the nascent substrate radical.^21,22^ This ensemble narrows and converges to a singular **S**_**2**_^**•**^ reactivity, as the progress coordinate through **S**_**1**_^**•**^ is traversed.^20^

**Figure 1:**
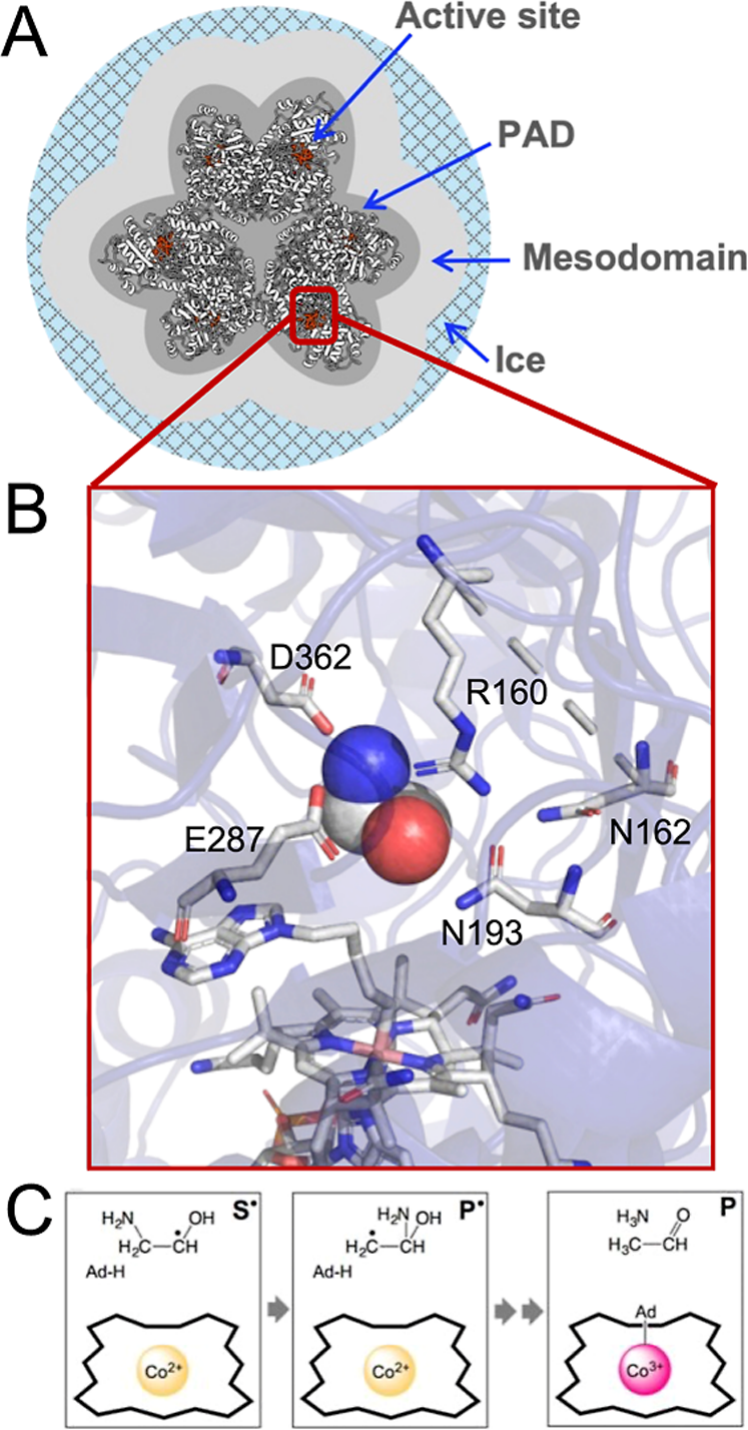
Depictions of the EAL enzyme,^16^ surrounding fluid phases, and core reaction sequence. (A) EAL in the low-temperature, frozen aqueous solution system. Bound adenosylcobalamin (red) locates the six active sites in each EAL oligomer. Radial direction of the two-dimensional cross section represents the relative mean dimensions for the condition of added 2% v/v dimethyl sulfoxide. PAD: protein-associated domain (hydration layer). (B) Protein-interior view of the active-site region, showing the substrate (van der Waals surface), active-site amino acids interacting directly with the substrate (labeled), and adenosylcobalamin (bottom). (C) Depiction of macrostates involved in the native substrate radical (**S** ^**•**^) decay reaction sequence through product radical (**P** ^**•**^) to diamagnetic bound products (**P**).

We have proposed that the kinetic bifurcation in the reaction of **S** ^**•**^ is correlated with a dynamical transition in the protein hydration solvent around EAL (protein-associated domain, PAD; Figure 1)^19^ that is detected by changes in EPR spin probe rotational motion^11,23^ and electric permittivity.^24^ This dynamical, or order–disorder transition (ODT; disorder-to-order, in the direction of decreasing *T*), is a general characteristic of the hydration solvent around folded proteins^25–28^ and is associated with the effective quenching of a particular class of cooperative, protein surface group-coupled solvent motions in the hydration layer,^29–31^ which have characteristics of the Johari–Goldstein-β class of local, collective cluster fluctuations,^32^ observed in a variety of hydrated materials.^33,34^ Below the ODT *T* value, the solvent and protein groups continue to execute thermal motions but only through incremental, individual fluctuations of the constituents of the clusters.^35^ A correlation of the *T* values of the ODT and kinetic bifurcation implies that the native reaction progress coordinate for EAL involves coupling of the motions of hydration solvent and active site groups, actuated by specific, or “select,” collective cluster fluctuations.^19^ Here, we test the proposed role of select configurational fluctuations in EAL by correlating the kinetic bifurcation with different ODT values established by different solvent conditions, including the standard aqueous-aminoethanol mesodomain system employed in the earlier kinetics measurements,^18,19^ and systems with added dimethyl sulfoxide, glycerol, and sucrose. The results show that the PAD ODT and kinetic bifurcation are matched in each system and, further, that the **S** ^**•**^, and **S**_**1**_^**•**^ and **S**_**2**_^**•**^, reaction kinetics adhere to a universal piecewise continuous Arrhenius profile, independent of solvent composition and absolute temperature. Our findings support the model^19^ that select configurational fluctuations, manifested at the active site by coupling with the hydration layer through the protein interior, contribute to the mechanism of substate transformations and chemical catalysis in EAL.

## Materials and Methods

### Materials

All chemicals used in the purification and sample preparation were purchased from commercial suppliers. Deionized water was used (resistivity, 18.2 MΩ·cm; Nanopure system, Siemens). All EPR samples were prepared in 4 mm-outer-diameter quartz EPR tubes (Wilmad-LabGlass, Buena, NJ, USA; P/N 707-SQ-250M). EPR samples were stored at 77 K.

### Protein Preparation and Purification

The EAL enzyme was obtained from an *Escherichia coli* overexpression system incorporating the *Salmonella typhimurium* EAL coding sequence,^36^ as described previously.^16,37^

### EPR Sample Preparation for TEMPOL Spin Probe Measurements

The standard, or control, EPR sample included 20 μM EAL (120 μM active sites), 100 mM ethanolamine (0.6% v/v), 10 mM potassium phosphate (pH 7.5), and 0.2 mM TEMPOL spin probe (4-hydroxy-TEMPO, Sigma-Aldrich; added from a freshly prepared stock solution in water), in a final volume of 0.3 mL. Samples were prepared and frozen in liquid nitrogen-chilled isopentane (140 K), as described.^23^ Other EPR samples contained, in addition to the standard constituents, dimethyl sulfoxide (DMSO, 2% v/v), glycerol (2% v/v), or sucrose (1.2% w/v), in a final volume of 0.3 mL.

### TEMPOL Spin Probe Measurements and Simulation Analysis

TEMPOL spin probe EPR spectra were obtained as a function of temperature and simulated to obtain component rotational correlation times and corresponding component amplitudes, or weights, by using the EasySpin toolbox in MATLAB (www.easyspin.org),^38^ as described in detail elsewhere.^23^

### EPR Sample Preparation for Permittivity Measurements

The standard, or control, EPR sample included 20 μM EAL (120 μM active sites), 100 mM 1-amino-2-propanol (0.7% v/v), 480 μM adenosylcobalamin, and 10 mM potassium phosphate (pH 7.5), in a final volume of 0.3 mL. The 1-amino-2-propanol substrate analog induces the cleavage of diamagnetic adenosyl-cob­(III)­alamin bound in EAL,^39^ forming bound paramagnetic (*S* = 1/2) cob­(II)­alamin, which is stable under anaerobic conditions. The EAL-bound cob­(II)­alamin can be trapped by freezing in liquid nitrogen-chilled isopentane (140 K), as described previously.^40^ Other EPR samples contained, in addition to the standard constituents, dimethyl sulfoxide (DMSO, 2% v/v), glycerol (2% v/v), or sucrose (1.2% w/v), in a final volume of 0.3 mL.

### Permittivity Measurements and Analysis

Measurements of the temperature-dependent amplitude of the EAL-bound cob­(II)­alamin EPR signal and analysis in terms of changes in sample electric permittivity have been described in detail^24^ and are summarized (Supporting Information, Appendix).

### EPR Sample Preparation for Substrate Radical Decay Measurements

The EAL enzyme was obtained from an *E. coli* overexpression system incorporating the *S. typhimurium* EAL coding sequence,^36^ as described previously.^16,37^ The standard, or control, EPR sample included 20 μM EAL (120 μM active sites), 480 μM adenosylcobalamin, 100 mM ethanolamine (0.6% v/v), and 10 mM potassium phosphate (pH 7.5) in a final volume of 0.3 mL. Samples were prepared on ice and frozen in liquid nitrogen-chilled isopentane (140 K), as described.^40^ Other EPR samples contained, in addition to the standard constituents, dimethyl sulfoxide (DMSO, 2% v/v), glycerol (2% v/v), or sucrose (1.2% w/v), in a final volume of 0.3 mL.

### Time-Resolved EPR Measurement and Analysis of the Substrate Radical Decay

Time-resolved, full-spectrum EPR spectroscopy was performed on a Bruker E500 ElexSys EPR spectrometer equipped with a Bruker ER4123 SHQE cavity.^40^ The cavity was equilibrated at the desired temperature and pretuned on a sample blank. After sample placement, the tune was adjusted, and the sample was allowed to come to the decay temperature (dead time, 30–60 s). Repetitive single-scan acquisition of EPR spectra (24 s sweep time; 2.56 ms time constant) continued throughout the duration of the decay. Decay spectra were processed and analyzed in MATLAB (MathWorks, Natick, MA).^19,40^

### Arrhenius Analysis of Temperature Dependence of Substrate Radical Decay Reaction Kinetics

The *T*-dependence of the first-order rate constant for substrate radical decay was assessed by using the molar form of the expression from Arrhenius rate theory^41^

1 $$k_{obs}=Ae^{\left({-E}_{a}/RT\right)}$$

where *A* (units, s^–1^), *E*_*a*_ (kcal/mol), and *R* (1.987 cal/mol/K) are the Arrhenius rate prefactor, activation energy, and gas constant, respectively. For the case that *E*_a_ and *A* are considered *T*-independent over the measurement range, plots of *k*_obs_ versus inverse *T* yield a linear relation with slope, $-\frac{E_{a}}{R}$ , and the vertical axis intercept of ln*A*

2 $${\ln k}_{obs}=\ln A-\frac{E_{a}}{RT}$$

## Results and Discussion

### Characterization of Temperature-Dependent Solvent Dynamics around EAL in the Different Solvent Systems by Using Spin Probe EPR Spectroscopy

The *T*-dependence of the protein-coupled solvent dynamics, including the ODT, was systematically varied by addition of dimethyl sulfoxide (2% v/v), glycerol (2% v/v), or sucrose (1.2% w/v) to the standard system used for measurement of the reaction kinetics,^19^ which includes EAL, buffer, and aminoethanol as substrates (100 mM or 0.6% v/v; designated as control). EPR spectra of the TEMPOL spin probe in these systems show the same general trend from low temperature (broad, rigid-limit line shape) to higher temperature (motionally narrowed line shape, characteristic of highly mobile spin probe) (Figure 2). However, the spectra display different absolute temperature dependences, as seen by comparison of the spectra across each row. The line shape differences are quantified by spectrum simulations^38^ (Figure 2, red overlaid curves). The simulations indicate contributions from two motional components with slow and fast rotational correlation times, τ_c,s_ and τ_c,f_, and corresponding normalized component weights, *W*_s_ and *W*_f_ (simulation parameters, Tables S1–S4). The temperature dependences of the τ_c_ (as the base-10 logarithm, logτ_c_) and *W* values are presented in Figure 3. As described previously for EAL in the presence of 0.5–4.0% v/v added dimethyl sulfoxide, the slow and fast mobility components correspond to TEMPOL that resides in the PAD hydration layer and the surrounding aqueous-cryosolvent mesodomain, respectively (Figure 1).^11,23^ The temperature dependences of the parameters display a range (Region III), where both correlation times lie within the mobility detection bandwidth (−10 ≤ logτ_c_ ≤ −7) and the constant *W*_s_ and *W*_f_ values reflect the relative volumes of disordered, fluid PAD, and mesodomain. An abrupt change in *W*_s_ and *W*_f_ values occurs at the Region II/III boundary, owing to the PAD ODT. The ordering of the PAD solvent leads to partial TEMPOL exclusion into the mesodomain,^11^ which increases the *W*_f_ values.

**Figure 2:**
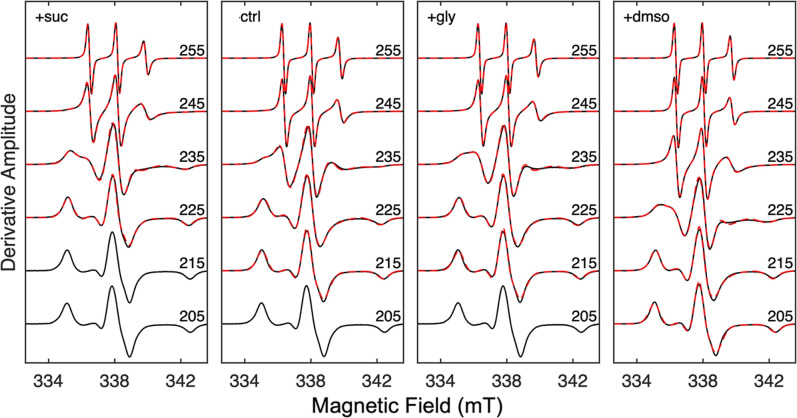
Temperature dependence of the TEMPOL EPR spectra (black), overlaid with simulations (red) for EAL in 0.6% v/v aminoethanol (ctrl), and with added 1.2% w/v sucrose (+suc), 2% v/v glycerol (+gly), and 2% v/v DMSO (+dmso). Spectra are labeled with acquisition *T* value (units, K). Absence of simulation indicates spin probe motion outside of the detection bandwidth. The spectra are normalized to the central peak-to-trough amplitude. Alignment along the magnetic field axis corresponds to the microwave frequency at 205 K.

**Figure 3:**
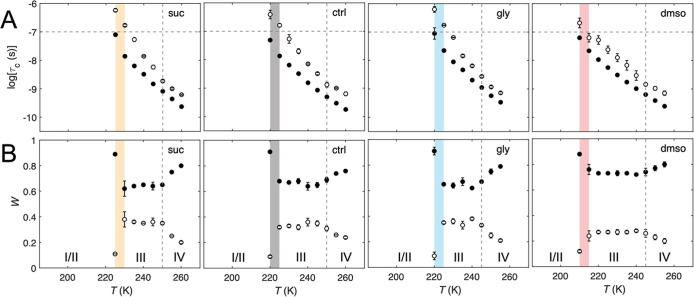
Temperature dependence of the rotational correlation time (A) and normalized mobility component weights (B) of TEMPOL for EAL and 0.6% v/v aminoethanol (ctrl) and with added 1.2% w/v sucrose (+suc), 2% v/v glycerol (+gly), and 2% v/v DMSO (+dmso). Solid symbols represent the fast component (logτ_c,f_, *W*_f_), and open symbols represent the slow component (logτ_c,s_, *W*_s_). The colored area indicates the ODT event for each system at the juncture of regions II and III. Vertical dashed lines distinguish Region III/IV of logτ_c_ behavior. The horizontal dashed line in (Α) represents the upper limit of logτ_c_ = – 7.0 for detection of TEMPOL motion. Error bars represent standard deviations from three separate determinations.

The ODT at 220 K for the control, aminoethanol-only system is shifted to a higher temperature by the added sucrose (225 K) and to a lower temperature by the added dimethyl sulfoxide (210 K) and remains essentially unchanged for glycerol addition (Figure 3). These ODT temperature differences reflect different degrees of confinement experienced by the PAD (increased confinement leads to an increased *T* value for the ODT),^11,23^ in the order from high to low: + sucrose > aminoethanol only ≈ + glycerol > + dimethyl sulfoxide. The different degrees of PAD confinement arise from differences in the coupled mesodomain solvent dynamics, which depend on the proximity of the PAD to the rigid ice boundary (governed by the thickness of the mesodomain) and intrinsic mesodomain solvent interactions.^11^

### Characterization of Temperature-Dependent Solvent Dynamics around EAL in the Different Solvent Systems by Using EPR-Detected Electric Permittivity

We used the solvent dielectric properties as an orthogonal approach for characterizing the *T*-dependence of the protein-coupled solvent dynamics and to gain insight into their mechanistic origin.^24^ Microwave absorption by the samples at the EPR frequency of 9.5 GHz is sensitive to solvent fluidity.^42^ Absorption of microwave energy by the rotating solvent dipoles lowers the loaded resonant cavity quality factor, *Q*_L_, through its dependence on the imaginary part of the complex electric permittivity, ε″. This decreases the EPR signal, *S*_T_ (Supporting Information, Appendix; Figure S1), in the limit of weak microwave power saturation (Figure S2). The EPR signal of the paramagnetic, low-spin (*S* = 1/2) Co­(II) in the cob­(II)­alamin cofactor bound in the protein interior in the active-site region of EAL was used as the sensor (Figure 4A).^24^ Figure 4B shows the increase in the temperature-normalized electric permittivity, *q*(ε″) (Supporting Information, Appendix), with increasing *T*, which is caused by the increase in thermal motions in the solvent around EAL.^42^ At the ODT, the change in the degree of solvent motion is reflected in a change from linear low to high *T* regimes of *q*(ε″) (Figure 4B). Significantly, the divergence from the low-*T* relations is consistent with the order of appearance of the spin probe-detected ODT *T* values in Figure 3.

**Figure 4:**
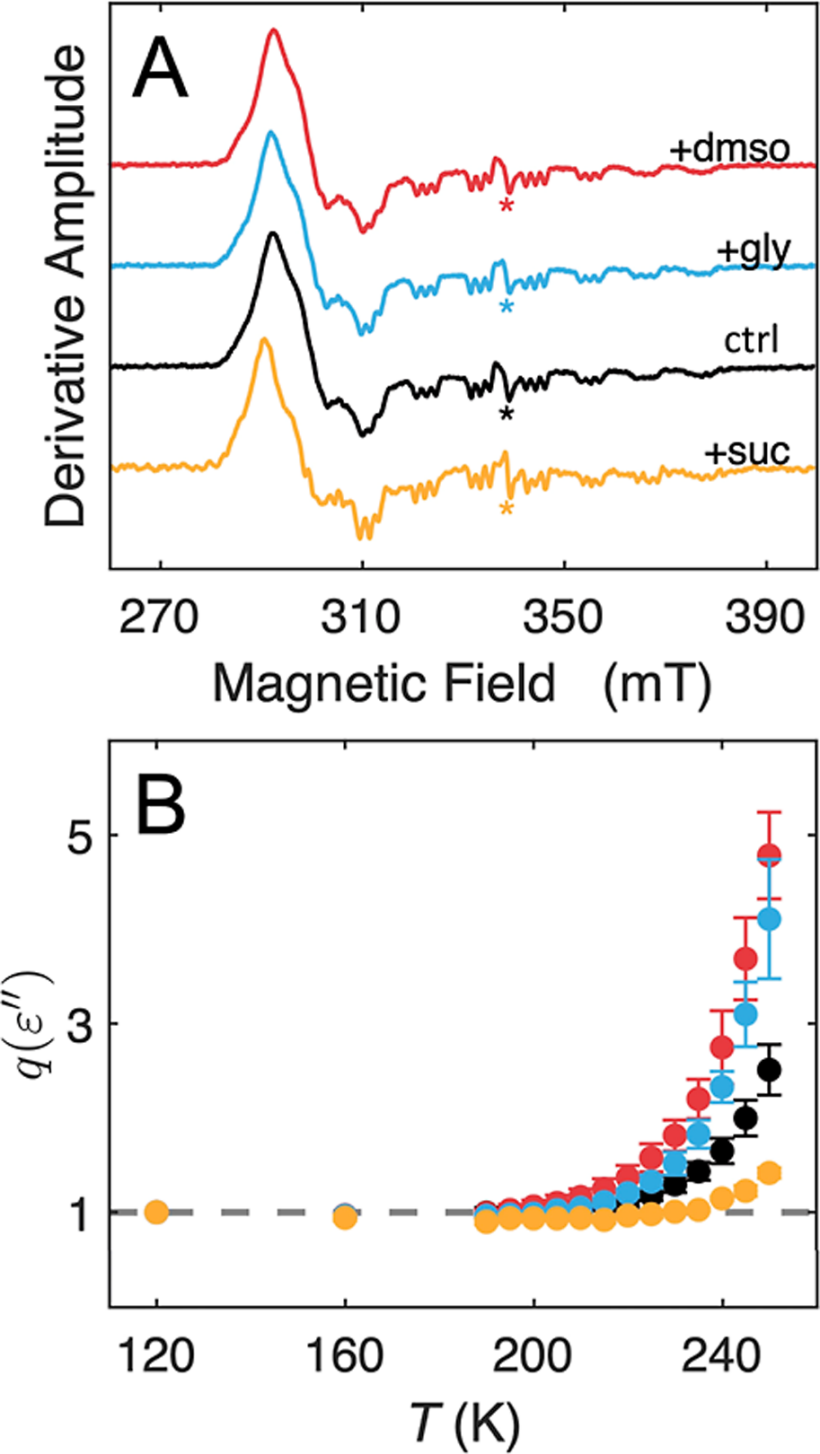
EPR spectra of cob­(II)­alamin bound in EAL and temperature dependence of sample permittivity, as the normalized loaded cavity quality factor, *q*(ε″). (A) EPR spectra of cob­(II)­alamin in EAL and 0.7% v/v 1-amino-2-propanol (ctrl), and with added 1.2% w/v sucrose (+suc), 2% v/v glycerol (+gly), and 2% v/v DMSO (+dmso), at 120 K. (B) Temperature dependences of *q*(ε″) with same color code as for panel A. EPR conditions: microwave frequency, 9.52 GHz; microwave power, 2.0 mW; magnetic field modulation, 1.0 mT; modulation frequency, 100 kHz; 8-scan average. *Minor impurity radical signal at *g* = 2.0.

The *T*-dependence of the solvent dielectric properties indicates a mechanistic origin of the ODT. The dielectric response at the relatively high, 9.5 GHz microwave frequency and the ODT in the temperature range of 200–240 K in each of the solvent systems (Figure 4) are consistent with an origin in the coupled motions of water and protein surface groups,^29–31^ as previously assigned.^24^ These motions have properties of the Johari–Goldstein-β class of fluctuations,^32–34^ which undergo a dynamical transition, with decreasing *T* value, from local, collective cluster motions to individual, incremental motions of the constituent groups. The local collective cluster fluctuations are distinct from lower frequency fluctuations^30,31^ of the large-scale collective α- and single-group β- classes.^43^

### Temperature Dependence of Substrate Radical Reaction Kinetics in the Different Solvent Systems

We measured the amplitude of the substrate radical component of the radical pair EPR spectrum as a function of time following temperature-step initiation of the decay reaction to diamagnetic products in each of the solvent systems (Figure 5A; first-order rate constant and amplitude parameters, Tables S6–S9). The monoexponential decay rate process (rate constant, *k*_obs,m_) prevailing at higher temperatures bifurcates at a characteristic lower temperature, leading to two first-order decays, slow (*k*_obs,s_; decay from **S**_**1**_^**•**^) and fast (*k*_obs,f_; **S**_**2**_^**•**^), that follow two distinct Arrhenius temperature dependences below the bifurcation temperature (Figure 5B–E). Therefore, the first-order rate constants for each solvent system preserve the characteristic piecewise-continuous pattern displayed by the control, aminoethanol-only system.^18,19^ However, the different solvent systems lead to a change in the absolute temperature of the kinetic bifurcation.

**Figure 5:**
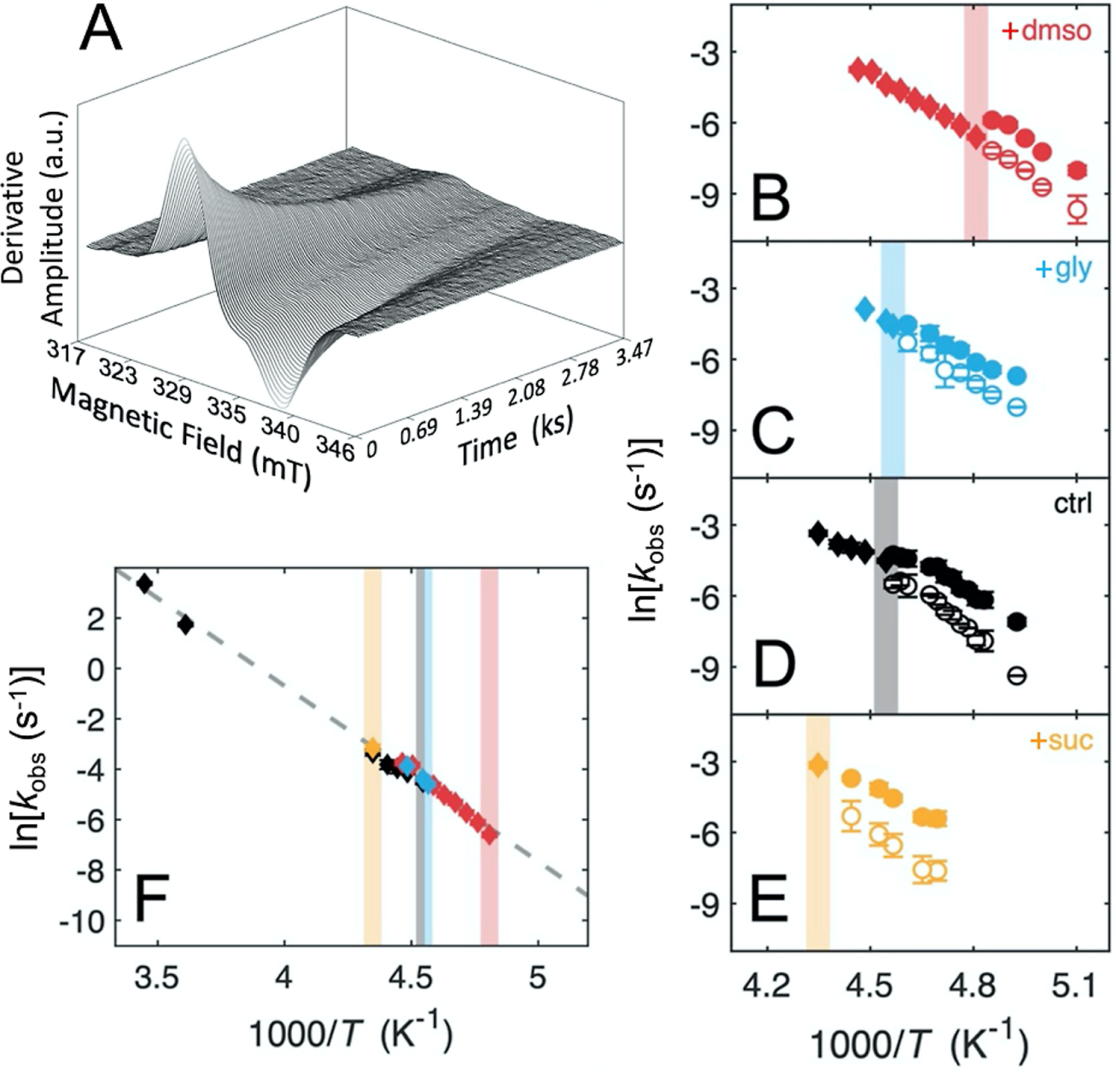
Temperature dependence of the substrate radical decay reaction. (A) Representative time dependence of the EPR spectrum of the substrate radical component following *T*-step reaction initiation (moving average over 5 spectra for 600 total spectra; EPR conditions and full radical pair spectrum, Figure S3; reproduced from ref 17, copyright 2017, American Chemical Society).^17^ (B–E) Arrhenius plots of observed first-order rate constants, for EAL and 0.6% v/v aminoethanol (ctrl),^17^ and with added 1.2% w/v sucrose (+suc), 2% v/v glycerol (+gly), and 2% v/v DMSO (+dmso). The colored vertical bar indicates the spin probe-detected ODT event for each system. For *T* above the bifurcation, *k*_obs,m_ (diamond) was obtained from monoexponential fitting. For *T* at or below the bifurcation temperature, *k*_obs,s_ (open circle) and *k*_obs,f_ (filled circle) were obtained from biexponential fitting. Standard deviations represent at least three separate decay measurements. (F) Arrhenius plot of only *k*_obs,m_ (*T* above bifurcation) with the same color code as for panels B–E and *k*_cat_ (277, 295 K)^17^ values. The dashed line represents the best linear fit to the data for all *k*_obs,m_ and *k*_cat_ (fit expression: ln*k* = – 6777/*T*+26.32; *R* ^2^ = 0.9943; corresponding Arrhenius parameters, Table S10).

### Connection of Temperature Dependence of the Substrate Radical Reaction Kinetics and Protein-Coupled Solvent Dynamics

As shown by the vertical bars in each panel of Figure 5B–E, the temperature of the bifurcation matches the temperature of the ODT for each solvent system. This provides compelling evidence that the ODT, and therefore the quenching of the collective cluster fluctuations in the protein-coupled hydration layer, is the origin of the kinetic bifurcation.

To reveal additional common features of the substrate radical reaction in EAL among the different solvent systems, we first compared the dependence of the monoexponential components (*k*_obs_ values above the ODT, *k*_obs,m_) on the inverse *T* (Figure 5F). The different systems adhere well to a singular linear dependence (*R* ^2^ = 0.9943; *R*, Pearson’s correlation coefficient), which includes the native reaction rate constants determined at 277 and 295 K in solution,^17^ with an *E*_a_ value of 13.5 ± 0.5 kcal/mol (Figure 5F; Arrhenius fitting parameters, Table S10). This common relation, which includes the room *T* values, provides evidence that the monoexponential decay (*k*_mono_) in each chemically distinct system proceeds by the native reaction mechanism. The linear fit in Figure 5F provides a foundation for comparison of the overall *T*-dependences of the decay kinetics under the different conditions. Thus, the different inverse *T*-dependences were translated along the monoexponential linear fit relation (Figure 5F) to match the bifurcation temperature points. The resulting plot shows a remarkable congruence of the log-rate-inverse *T* relations, including the *k*_obs,s_ and *k*_obs,f_ values (Figure 6). This congruency plot indicates that the dependence of the **S**_**1**_^**•**^ and **S**_**2**_^**•**^ substate interconversion and reaction steps on the protein-coupled solvent fluctuations is relatively insensitive to the chemical composition of the mesodomain solvent and the absolute *T* value and therefore depends primarily on the physical properties of the fluctuations.

### Select Configurational Fluctuation Dependence of S_1_^•^ and S_2_^•^Substate Interconversion and Chemical Reactions

The correlation of the quenching of the collective cluster fluctuations at the ODT with the kinetic bifurcation establishes that configurational conversion of the capture substate, **S**_**1**_^**•**^, to the reaction-enabled substate, **S**_**2**_^**•**^, is coupled obligatorily to the collective cluster fluctuations in the PAD. The primacy of a change in fluctuations, or dynamics, rather than a structural change, is supported by the general absence of significant equilibrium structure change for dynamical transitions,^43,44^ as well as by the following observations in the EAL system: (1) the Arrhenius relation across the bifurcation is piecewise continuous, rather than discontinuous. Discontinuity is expected for a significant structure change. (2) The cob­(II)­alamin-substrate radical pair EPR line shape, which is sensitive to spin–spin distance changes of ∼1 Å,^45,46^ is not altered across the bifurcation. (3) The EPR line shape of the substrate radical component of the weakly coupled radical pair in EAL, which is sensitive to the substrate radical conformation,^45,47^ is preserved across the bifurcation and is independent of decay time. Overall, the results show a select dynamical coupling of the PAD collective cluster fluctuations to the active site fluctuations that actuate interconversion of the **S**_**1**_^**•**^ and **S**_**2**_^**•**^ substates.

To identify the possible contribution of select dynamical coupling to the chemical reaction steps, we compared the *E*_a_ values for the common monoexponential decay (Figure 5 F; *E*_a_ = 13.5 ± 0.5 kcal/mol) and the bifurcation component decays (Arrhenius plots and fitting parameters, Figure S4 and Table S10). For each solvent condition, the *E*_a_ values increase as *T* decreases across the ODT. The mean values of *E*_a_ for all conditions are 21.0 ± 3.8 kcal/mol for the slow component and 16.7 ± 2.2 kcal/mol for the fast component. The corresponding mean increase in *E*_a_ for **S**_**1**_^**•**^ relative to **S** ^**•**^ decay is 7.5 ± 3.8 kcal/mol, while the mean increase in *E*_a_ for **S**_**2**_^**•**^ relative to **S** ^**•**^ decay is 3.2 ± 2.3 kcal/mol. Therefore, coupling to PAD collective cluster fluctuations contributes significantly to the active site configurational changes involved in the chemical reactions from **S**_**1**_^**•**^ and **S**_**2**_^**•**^. In particular, the select coupling to the native chemical reaction of the **S**_**2**_^**•**^ substate contributes approximately one-quarter of the lowering of the activation energy barrier.

The role of select fluctuations in the native catalytic sequence is also supported by results from an engineered, non-native chemical reaction in EAL,^48^ for which the Arrhenius relation remains linear through the ODT. This indicates an absence of select PAD collective cluster coupling and, thus, that the non-native reaction is coupled to the generic background fluctuations. Select fluctuations are also consistent with the microstate ensemble character of certain enzyme intermediate states,^5^ and the ensemble-function characterization of enzyme catalysis,^7^ in showing how the system moves, selectively, through the configuration space to achieve both catalysis and fidelity.^49^ Our results imply a coupling of the PAD fluctuations, through the protein, to the active site, in agreement with effects of protein surface mutations on active site events^50^ and mechanistic proposals.^51–53^ The identification of intraprotein conduction pathway(s) for select fluctuations is a future challenge in elaborating the dynamical basis of enzyme catalysis in EAL.

**Figure 6:**
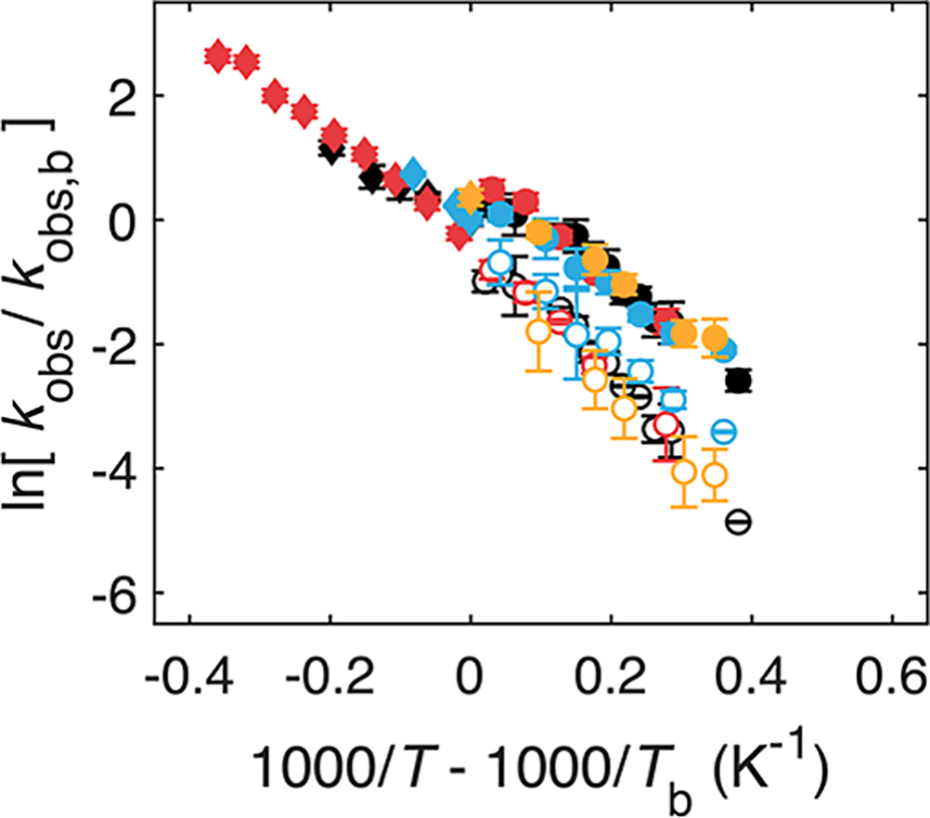
Reduced Arrhenius plots of observed first-order rate constants from all samples. Sample conditions correspond to EAL and 0.6% v/v aminoethanol (black),^17^ and with added 1.2% w/v sucrose (gold), 2% v/v glycerol (blue), and 2% v/v DMSO (red). Arrhenius relations are aligned to their bifurcation points (1000/*T*_b_, ln­[*k*_obs,b_]), by diagonal translation along the *k*_obs,m_ fit segment of the Arrhenius relation, shown by the dashed line in Figure 5F.

## Conclusions

Reactions at enzyme active sites generally do not follow the spatial progression of the assembly line paradigm. Rather, the complex manipulations occur in the same location in space, in a temporal progression. In the case of EAL, this requires radical intermediate stabilization, actuation, bond-breaking, and bond-making steps, conducted by interactions with a constant repertoire of amino acid side chain, backbone, and bound water structural groups at the active site. The select coupling of PAD hydration layer configurational fluctuations of the collective cluster class to the protein reconfiguration among **S**_**1**_^**•**^ and **S**_**2**_^**•**^substates in the active site of EAL indicates the existence of a degree of synchrony in the sequence of thermally driven, stochastic motions of active site groups that actuate the substate transition. The vast reservoir of random single-group and other collective thermal fluctuations^8,9^ is not sufficient to guide the highly reactive radical species through the **S**_**1**_^**•**^ to **S**_**2**_^**•**^ substates along the progress coordinate for catalysis. Select PAD solvent collective cluster fluctuations also couple significantly to the chemical reaction of the **S**_**2**_^**•**^substate, contributing approximately one-fourth of the native lowering of the activation energy barrier. Select fluctuations, representing a specific contribution of dynamics as resolved from structure, are an essential feature in the description of EAL catalysis, and we propose, make fundamental contributions to the catalytic prowess of enzymes, in general.

## Supplementary Material



## Notes

Appendix describing analysis of the temperature-dependent electric permittivity of the EPR samples; plot of the dependence of the EPR signal amplitude of EAL-bound cob­(II)­alamin on inverse temperature for the different solvent systems; plot of the microwave power dependence of the EPR amplitude of EAL-bound cob­(II)­alamin at different temperature values; plot of the cob­(II)­alamin-substrate radical pair EPR spectrum; Arrhenius plots and best-fit linear relations for the observed biexponential first-order rate constants obtained for the substrate radical EPR signal decay, in the different solvent systems; tables of the mean TEMPOL logτ_c_ and *W* values at different *T* values for the different solvent systems; table of values for temperature dependence of the normalized low-field peak amplitude of cob­(II)­alamin in the active site of EAL for the different solvent systems; table of observed first-order rate constants and normalized amplitude parameters for cob­(II)­alamin-substrate radical pair decay kinetics at different temperatures for the different solvent systems; and table of activation enthalpy and entropy values obtained from Arrhenius analysis of the microscopic rate constants; references (PDF)

## Abbreviations

EAL: ethanolamine ammonia-lyase
EPR: electron paramagnetic resonance
ODT: order–disorder transition
PAD: protein-associated domain
DMSO: dimethyl sulfoxide
