# Supplementary material for: Select Configurational Dynamics in Ethanolamine Ammonia-Lyase Radical Enzyme Catalysis
Source: ACS Phys Chem Au. 2025 Sep 2;5(6):609–17. doi: 10.1021/acsphyschemau.5c00051 (PMC12670283; doi:10.1021/acsphyschemau.5c00051)
Supplement: Supplementary file 1 [file pg5c00051_si_001.pdf]

Supporting Information for:

Select Configurational Dynamics in Ethanolamine Ammonia-Lyase Radical Enzyme Catalysis

Wei Li, Andrew M. Stewart, and Kurt Warncke*

Department of Physics, Emory University, Atlanta, GA 30322

* Corresponding author: kwarncke@physics.emory.edu

Contents

Appendix

Page

Analysis of the Temperature-Dependent Electric Permittivity of cob(II)alamin-EAL EPR samples. S4

Figures

Figure S1. Dependence of the EPR signal amplitude of EAL-bound cob(II)alamin on inverse temperature for the different solvent systems. S7

Figure S2. Microwave power dependence of the EPR amplitude of EAL-bound cob(II)alamin at different temperature values. S8

Figure S3. Cob(II)alamin-substrate radical radical pair EPR spectrum in EAL, generated by using aminoethanol. S9

Figure S4. Arrhenius plots and best-fit linear relations for the observed mono- and bi-exponential first-order rate constants obtained for the substrate radical EPR signal decay, for the different cryosolvent systems. S10

Tables

Table S1. Mean TEMPOL logτ_c and W values at different T values: aminoethanol (control). S11

Table S2. Mean TEMPOL logτ_c and W values at different T values: aminoethanol, with added sucrose. S12

Table S3. Mean TEMPOL logτ_c and W values at different T values: aminoethanol, with added glycerol. S13

Table S4. Mean TEMPOL logτ_c and *W* values at different *T* values: aminoethanol, with added dimethylsulfoxide. S14

Table S5. Temperature dependence of the normalized low-field peak amplitude of cob(II)alamin in the active site of EAL in frozen aqueous solution with 1-amino-2-propanol (control), and with different added cryosolvents. S15

Table S6. Observed first-order rate constants and normalized amplitude parameters for cob(II)alamin-substrate radical pair decay kinetics at different temperatures: aminoethanol (control). S16

Table S7. Observed first-order rate constants and normalized amplitude parameters for cob(II)alamin-substrate radical pair decay kinetics at different temperatures: aminoethanol, with added sucrose. S17

Table S8. Observed first-order rate constants and normalized amplitude parameters for cob(II)alamin-substrate radical pair decay kinetics at different temperatures: aminoethanol, with added glycerol. S18

Table S9. Observed first-order rate constants and normalized amplitude parameters for cob(II)alamin-substrate radical pair decay kinetics at different temperatures: aminoethanol, with added dimethylsulfoxide. S19

Table S10. Activation enthalpy and entropy values obtained from Arrhenius analysis of the microscopic rate constants. S20

References S21

Appendix

Analysis of the Temperature-Dependent Electric Permittivity of Cob(II)alamin-EAL EPR Samples

This Appendix describes the dependence of the EPR signal on temperature (*T*) and sample electric permittivity (dielectric), toward development of an expression for the *T*-dependence of sample electric permittivity, isolated from the common inverse-*T* dependence (Curie law) among different samples. This is valuable for the characterization of the *T*-dependence change in electric permittivity, that represents a change in the solvent dynamics around the protein, and in particular, the effect of the order-disorder transition in the hydration solvent.[1]

The general expression for the CW EPR signal intensity, *S*, of a static electron is:

$$S = \chi'' \eta Q_L \sqrt{P Z_0} \quad \text{(S1)}$$

where χ'' is the imaginary component of the effective magnetic susceptibility, dimensionless η is the resonator filling factor, Q_L is the loaded quality factor of the resonator (with quartz Dewar insert and sample, under the experimental conditions), P is the microwave power to the resonator produced by the external microwave source, and Z_0 is the characteristic impedance of the transmission line.[2-4] By solving the dynamical Bloch equations, χ'' of an electron in an external magnetic field and interacting with microwave radiation takes the form:

$$\chi'' = \frac{\chi_0 \gamma_e H_0 T_2}{1 + (\gamma_e H_0 - \omega)^2 T_2^2 + \gamma_e^2 H_1^2 T_1 T_2} \quad \text{(S2)}$$

where χ_0 is the volume magnetic susceptibility, γ_e is the electron gyromagnetic ratio, T_1 and T_2 are the spin-lattice and spin-spin relaxation time constants, H_0 and H_1 are the static and microwave magnetic fields, and ω is the microwave frequency.[3-5] The term $\gamma_e^2 H_1^2 T_1 T_2$ represents the microwave power saturation effect and decreases the observed signal. At resonance, $\gamma_e H_0 \rightarrow \omega$, and in the absence of power saturation effect, corresponding to $\gamma_e^2 H_1^2 T_1 T_2 \ll 1, \chi''$ is reduced to:

$$\chi'' = \chi_0 H_0 \gamma_e T_2 \text{S3)}$$

Curie's law gives an explicit expression for χ_0 for a spin 1/2 system, as follows:

$$\chi_0 = \frac{\mu N_e \hbar^2 \gamma_e^2}{4 k_B T} \text{(S4)}$$

where μ is the magnetic permeability, N_e is the unpaired electron density of the sample, and $\hbar$ is the reduced Planck's constant.

The quality factor, Q, indicates the efficiency of the cavity in storing microwave energy.[2-4] Contributions to Q_L from the sample absorbance at resonance and surface currents at the sample are negligible, relative to the effect of dielectric losses in the cavity and sample.[2-4] Consequently, for a critically coupled resonator, Q_L is a function of only the unloaded quality factor, Q_U (resonator in isolation), and the imaginary part of the complex electric permittivity, ε'', as follows:

$$\frac{1}{Q_L} = \frac{2}{Q_U} + \frac{\pi r^3}{a V_c} \text{(S5)}$$

where r is the radius of the cylindrical EPR sample, a is the dimension of the cavity coaxial with the cylindrical sample, and V_c is the volume of the empty cavity resonator.[4, 6] The equations above lead to the following expression for the unsaturated EPR signal at resonance:

$$S = \frac{\mu N_e \hbar^2 \gamma_e^3}{4k_B T} H_0 T_2 \eta Q_L(\varepsilon") \sqrt{PZ_0} = \frac{C}{T} Q_L(\varepsilon") \sqrt{P} (S6)$$

where C is a constant. Overall, below saturation, the EPR signal amplitude is proportional to $1/T$ and $\sqrt{P}$, and it has an anti-correlation with the dielectric losses of the sample [$Q_L(\varepsilon")$ decreases with increase in microwave absorption by the sample].

To obtain an expression for comparison of the dielectric properties of different samples, that does not include the intrinsic, $1/T$ dependence from Curie's law, that is common to all samples, we introduce the relative loaded quality factor, $q(\varepsilon")$, as follows:

$$q(\varepsilon") = \frac{Q_{L,\,120\,K}(\varepsilon")}{Q_{L,T}(\varepsilon")} = \frac{S_{120\,K} \times 120\ K}{S_T \times T} = 1 + \frac{\pi r^3 Q_U}{2aV_c} (S7)$$

Equation S7 includes normalization of the signal, S_T, at temperature, T, to the signal at 120 K, $S_{120\,K}$, under the assumption that $\varepsilon"$ approaches a common, negligible value at 120 K for the frozen aqueous-cosolvent EPR samples examined in the present work. Equation S7 expresses that thermal motion in the the sample at temperature, T, caused by absorption of microwave energy, lowers $Q_{L,T}(\varepsilon")$ relative to the reference value, $Q_{L,120\,K}(\varepsilon")$, which causes an increase in $q(\varepsilon")$.

Figures

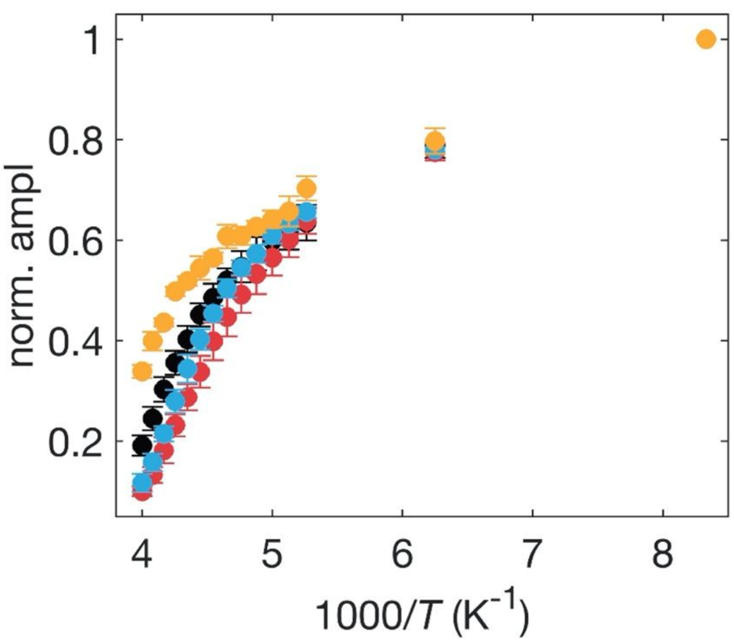

Figure S1. Dependence of the EPR signal amplitude of EAL-bound cob(II)alamin on inverse temperature for the different solvent systems. The vertical axis represents the signal amplitude, *S*, normalized to the value at 120 K, for each solvent system. The vertical axis is proportional to the relative (normalized) loaded cavity quality factor, as defined in Eqn. S7. Linear regions at the low and high temperature regions correspond to effectively uniform solvent physical properties. Spectra were obtained under anaerobic conditions in the presence of 0.7% v/v 1-amino-2-propanol (control, black), with added 1.2% w/v sucrose (gold), 2% w/v glycerol (blue) or 2% v/v DMSO (red). *EPR conditions*: microwave frequency, 9.52 GHz; microwave power, 2.0 mW; magnetic field modulation, 1.0 mT; modulation frequency, 100 kHz; temperature, 120 K; 8-scan average.

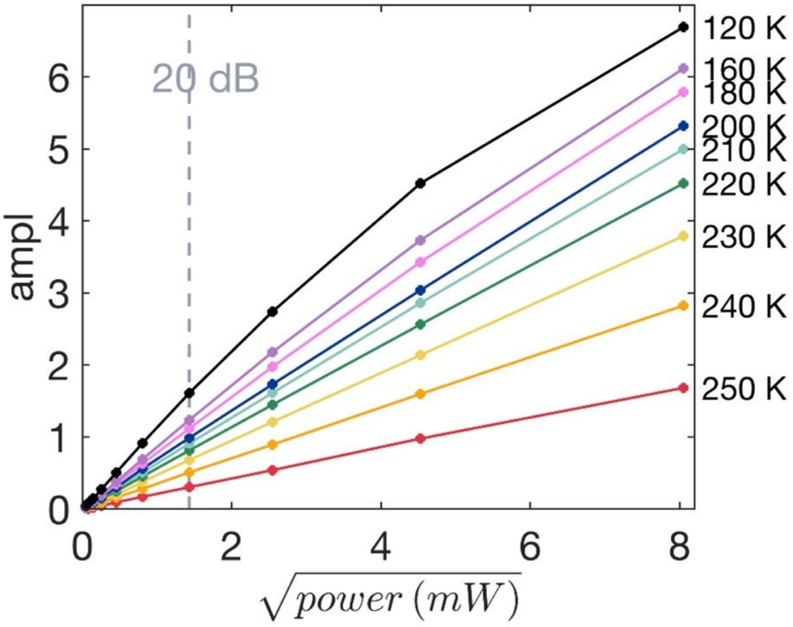

Figure S2. Microwave power dependence of the EPR amplitude of EAL-bound cob(II)alamin at different temperature values. Grey dashed line shows the microwave power of 2 mW (20 dB attenuation of full microwave power) used for collection of the T-dependence experiments. Linearity is observed through the power level of 2 mW for all temperature values, indicating that the measurements of loaded cavity quality factor, Q_L, based on the EPR signal, S, are not impacted by microwave power saturation at the standard EPR acquisition power of 2 mW. Spectra were obtained under anaerobic conditions. *EPR conditions*: microwave frequency, 9.52 GHz; magnetic field modulation, 1.0 mT; modulation frequency, 100 kHz; 8-scan average.

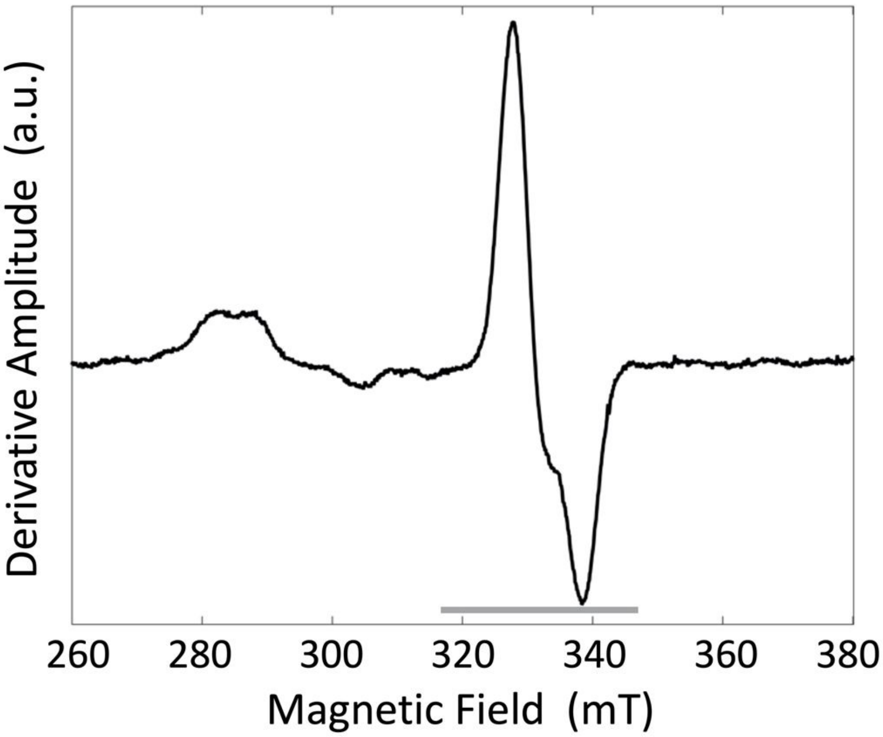

Figure S3. Cob(II)alamin-substrate radical pair EPR spectrum in EAL, generated by using aminoethanol.[7] Grey bar shows region dominated by the substrate radical, as presented in Figure 5A, that is used to obtain the amplitude in kinetics measurements (peak-to-trough amplitude). The amplitude from Co(II) in cob(II)alamin (electron spin, S=1/2) manifests most strongly in the $g_{\perp}$ region around 270-290 mT. Spectrum reproduced from reference 7, copyright 2017, American Chemical Society. *EPR conditions*: microwave frequency, 9.34 GHz; microwave power, 20 mW; magnetic field modulation, 1.0 mT; modulation frequency, 100 kHz; temperature, 207 K; single scan.

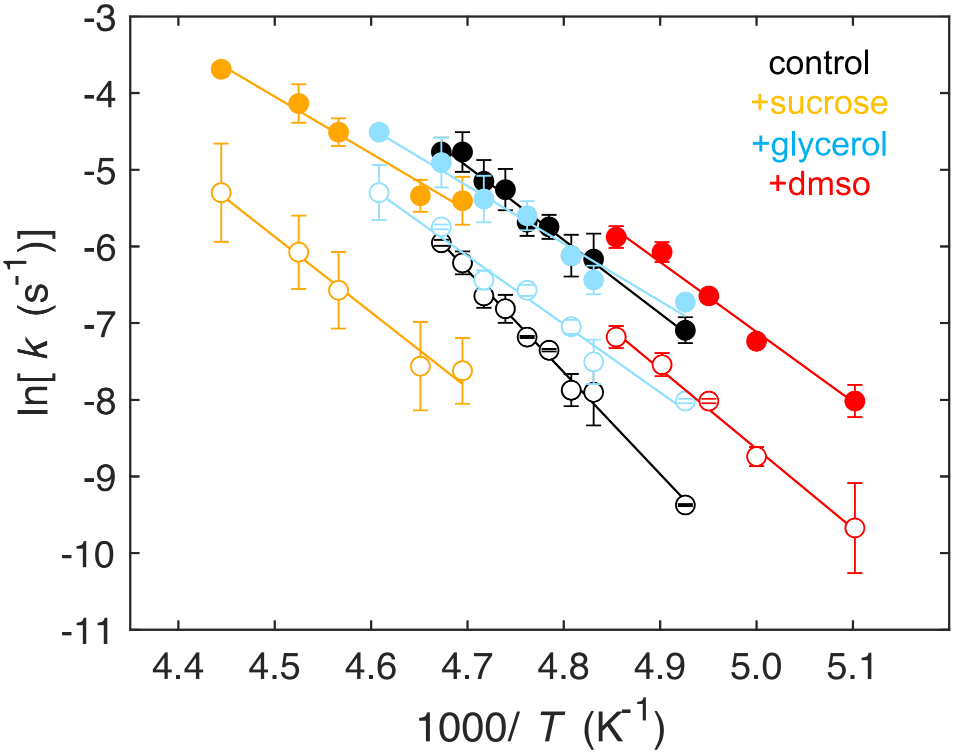

Figure S4. Arrhenius plots and best-fit linear relations for the observed bi-exponential first-order rate constants obtained for the substrate radical EPR signal decay, in the different cryosolvent systems. The mean rate constant values for the slow decay component (o) and the fast decay component (•) for each temperature are plotted, and error bars represent the standard deviation for at least three separate decay measurements. Conditions include 0.6% v/v aminoethanol, (control, ctrl, black), and with added 1.2% w/v sucrose (+sucrose, gold), 2% v/v glycerol (+glycerol, blue), and 2% v/v dimethylsulfoxide (+dmso, red). Fitting parameters are presented in Table S10.

Tables

Table S1. TEMPOL logτ_c and W values at different T values for 100 mM aminoethanol (control), without cryosolvent.

T (K)	log $\tau_{c,s}$ (s)	W_s	log $\tau_{c,f}$ (s)	W_f
215	-5.77 ± 0.03	0.09 ± 0.03	-6.70 ± 0.05	0.91 ± 0.03
220	-6.41 ± 0.15	0.09 ± 0.01	-7.31 ± 0.03	0.91 ± 0.01
225	-6.79 ± 0.06	0.32 ± 0.01	-7.87 ± 0.04	0.68 ± 0.01
230	-7.27 ± 0.15	0.33 ± 0.01	-8.19 ± 0.00	0.67 ± 0.01
235	-7.70 ± 0.09	0.32 ± 0.02	-8.49 ± 0.06	0.68 ± 0.02
240	-8.15 ± 0.03	0.36 ± 0.03	-8.81 ± 0.03	0.64 ± 0.03
245	-8.49 ± 0.04	0.35 ± 0.02	-9.07 ± 0.02	0.65 ± 0.02
250	-8.88 ± 0.09	0.31± 0.02	-9.31 ± 0.01	0.69 ± 0.02
255	-9.00 ± 0.02	0.27 ± 0.00	-9.53 ± 0.01	0.73 ± 0.00
260	-9.20 ± 0.07	0.24 ± 0.01	-9.75 ± 0.01	0.76 ± 0.01

Table S2. TEMPOL logτ_c and W values at different T values for 100 mM aminoethanol, with 1.2% w/v added sucrose cryosolvent.

T (K)	log $\tau_{c,s}$ (s)	W_s	log $\tau_{c,f}$ (s)	W_f
220	-5.54 ± 0.08	0.08 ± 0.01	-6.46 ± 0.05	0.92 ± 0.01
225	-6.24 ± 0.05	0.11 ± 0.00	-7.10 ± 0.06	0.89 ± 0.00
230	-6.77 ± 0.04	0.38 ± 0.06	-7.86 ± 0.06	0.62 ± 0.06
235	-7.27 ± 0.07	0.36 ± 0.01	-8.20 ± 0.02	0.64± 0.01
240	-7.86 ± 0.02	0.35 ± 0.00	-8.49 ± 0.01	0.65 ± 0.00
245	-8.24 ± 0.07	0.36 ± 0.03	-8.83 ± 0.02	0.64 ± 0.03
250	-8.73 ± 0.04	0.35 ± 0.01	-9.09 ± 0.01	0.65 ± 0.01
255	-9.00 ± 0.03	0.25 ± 0.00	-9.36 ± 0.00	0.75 ± 0.00
260	-9.21 ± 0.02	0.20 ± 0.01	-9.63 ± 0.01	0.80 ± 0.01

Table S3. TEMPOL logτ_c and W values at different T values for 100 mM aminoethanol, with 2% v/v added glycerol cryosolvent.

T (K)	log $\tau_{c,s}$ (s)	W_s	log $\tau_{c,f}$ (s)	W_f
220	-6.16 ± 0.00	0.08 ± 0.03	-6.67 ± 0.03	0.92 ± 0.03
225	-6.76 ± 0.01	0.35 ± 0.01	-7.65 ± 0.05	0.65 ± 0.01
230	-7.19 ± 0.01	0.36 ± 0.02	-8.05 ± 0.00	0.64 ± 0.02
235	-7.81± 0.04	0.33 ± 0.03	-8.33 ± 0.02	0.67± 0.03
240	-8.19 ± 0.04	0.38 ± 0.01	-8.69 ± 0.01	0.62 ± 0.01
245	-8.56 ± 0.04	0.33 ± 0.01	-8.95 ± 0.01	0.67 ± 0.01
250	-8.93 ± 0.04	0.25 ± 0.02	-9.24 ± 0.05	0.75 ± 0.02
255	-9.14± 0.04	0.21 ± 0.01	-9.47 ± 0.01	0.79 ± 0.01

Table S4. TEMPOL logτ_c and W values at different T values for 100 mM aminoethanol, with 2% v/v added dimethylsulfoxide (DMSO) cryosolvent.

T (K)	log $\tau_{c,s}$ (s)	W_s	log $\tau_{c,f}$ (s)	W_f
205	-6.12 ± 0.10	0.09 ± 0.01	-6.71 ± 0.09	0.91 ± 0.01
210	-6.68 ± 0.17	0.12 ± 0.01	-7.20 ± 0.04	0.88 ± 0.01
215	-6.96 ± 0.15	0.24 ± 0.04	-7.66 ± 0.03	0.76 ± 0.04
220	-7.28 ± 0.15	0.27 ± 0.01	-7.97 ± 0.02	0.73 ± 0.01
225	-7.62 ± 0.13	0.27 ± 0.01	-8.25 ± 0.01	0.73 ± 0.01
230	-7.90 ± 0.14	0.27 ± 0.02	-8.51 ± 0.01	0.73 ± 0.02
235	-8.17 ± 0.17	0.27 ± 0.01	-8.76 ± 0.01	0.73 ± 0.01
240	-8.53 ± 0.17	0.28 ± 0.01	-8.99 ± 0.01	0.72 ± 0.01
245	-8.84 ± 0.06	0.26 ± 0.03	-9.20 ± 0.00	0.74 ± 0.03
250	-8.98 ± 0.07	0.23 ± 0.02	-9.41 ± 0.00	0.77 ± 0.02
255	-9.15 ± 0.08	0.20 ± 0.02	-9.41 ± 0.01	0.80 ± 0.02

Table S5. Temperature dependence of the normalized low-field peak amplitude of the cob(II)alamin $g_{\perp}$ feature under anaerobic conditions in the active site of EAL in frozen aqueous solution with 100 mM 1-amino-2-propanol (control), and with different added cryosolvents.

***T* (K)**	**1.2% w/v sucrose**	**control**	**2% v/v glycerol**	**2% v/v dmso**
120	1	1	1	1
160	0.80 ± 0.03	0.78 ± 0.04	0.78 ± 0.01	0.78 ± 0.02
190	0.70 ± 0.02	0.63 ± 0.04	0.66 ± 0.01	0.64 ± 0.03
195	0.66 ± 0.03	0.62 ± 0.03	0.63 ± 0.01	0.60 ± 0.03
200	0.64 ± 0.02	0.60 ± 0.03	0.61 ± 0.02	0.57 ± 0.04
205	0.63 ± 0.01	0.57 ± 0.03	0.57 ± 0.02	0.53 ± 0.04
210	0.61 ± 0.02	0.55 ± 0.02	0.55 ± 0.01	0.49 ± 0.04
215	0.61 ± 0.02	0.52 ± 0.03	0.50± 0.02	0.45 ± 0.04
220	0.56 ± 0.01	0.49 ± 0.02	0.45± 0.02	0.40 ± 0.04
225	0.54 ± 0.02	0.45 ± 0.03	0.40 ± 0.02	0.34 ± 0.03
230	0.52 ± 0.01	0.40 ± 0.02	0.34 ± 0.03	0.30 ± 0.03
235	0.50 ± 0.01	0.36 ± 0.03	0.28 ± 0.02	0.13 ± 0.02
240	0.44 ± 0.01	0.30 ± 0.02	0.21 ± 0.02	0.18 ± 0.03
245	0.40 ± 0.02	0.24 ± 0.02	0.16 ± 0.02	0.13 ± 0.02
250	0.34 ± 0.01	0.19 ± 0.02	0.12 ± 0.02	0.10 ± 0.01

Table S6. Observed first-order rate constant and normalized amplitude parameters for the fit of the mono- and biexponential functions to the substrate radical decay kinetics at different temperatures for 100 mM aminoethanol (control).[7] Data reproduced from reference 7, copyright 2017, American Chemical Society.

T (K)	$k_{obs,f}$ (s^{-1})	$A_{obs,f}$	$k_{obs,s}$ (s^{-1})	$A_{obs,s}$	R^2
203	8.3 (±1.4) ×10^{-4}	0.60 ± 0.03	8.5(±0.1) ×10^{-5}	0.40 ± 0.03	0.9978
207	2.1 (±0.7) ×10^{-3}	0.46 ± 0.16	3.7 (±1.6) ×10^{-4}	0.54 ± 0.16	0.9993
208	2.2 (±0.6) ×10^{-3}	0.49 ± 0.12	3.8 (±0.8) ×10^{-4}	0.51 ± 0.12	0.9988
209	3.2(±0.5) ×10^{-3}	0.38 ± 0.08	6.4 (±0.1) ×10^{-4}	0.62 ± 0.08	0.9992
210	3.4 (±0.6) ×10^{-3}	0.39 ± 0.07	7.6 (±0.1) ×10^{-4}	0.61 ± 0.07	0.9992
211	5.2 (±1.4) ×10^{-3}	0.30 ± 0.15	1.1 (±0.2) ×10^{-3}	0.70 ± 0.15	0.9986
212	5.8 (±1.6) ×10^{-3}	0.33 ± 0.13	1.3 (±0.2) ×10^{-3}	0.67 ± 0.13	0.9993
213	8.5 (±2.2) ×10^{-3}	0.30 ± 0.13	2.0 (±0.3) ×10^{-3}	0.70 ± 0.13	0.9992
214	8.5 (±1.6) ×10^{-3}	0.32 ± 0.10	2.6 (±0.1) ×10^{-3}	0.68 ± 0.10	0.9993
217	1.2 (±0.4) ×10^{-2}	0.63 ± 0.09	3.8 (±1.8) ×10^{-3}	0.37 ± 0.09	0.9990
218	1.3 (±0.2) ×10^{-2}	0.56 ± 0.09	4.6 (±0.8) ×10^{-3}	0.44 ± 0.09	0.9953
219	1.4 (±0.1) ×10^{-2}	0.62 ± 0.12	4.1 (±0.7) ×10^{-3}	0.38 ± 0.12	0.9945
220	1.1 (±0.1) ×10^{-2}	1			0.9978
223	1.6 (±0.1) ×10^{-2}	1			0.9982
225	1.9 (±0.4) ×10^{-2}	1			0.9748
227	2.2 (±0.4) ×10^{-2}	1			0.9450
230	3.5 (±0.4) ×10^{-2}	1			0.9460

Table S7. Observed first-order rate constant and normalized amplitude parameters for the fit of the mono- and biexponential functions to the substrate radical decay kinetics at different temperatures for 100 mM aminoethanol, with 1.2% w/v added sucrose cryosolvent.

T **(K)**	$k_{obs,f}$ **(s^{-1})**	$A_{obs,f}$	$k_{obs,s}$ **(s^{-1})**	$A_{obs,s}$	R^2
213	4.5 (±1.4) ×10^{-3}	0.52 ± 0.05	4.9 (±2.1) ×10^{-4}	0.48 ± 0.05	0.9949
215	4.8 (±1.0) ×10^{-3}	0.60 ± 0.06	5.2 (±3.0) ×10^{-4}	0.40 ± 0.06	0.9937
219	1.1 (±0.2) ×10^{-2}	0.61 ± 0.04	1.4 (±0.7) ×10^{-3}	0.39 ± 0.04	0.9969
221	1.6 (±0.4) ×10^{-2}	0.63 ± 0.08	2.3 (±1.1) ×10^{-3}	0.27 ± 0.08	0.9984
225	2.5 (±0.2) ×10^{-2}	0.74 ± 0.08	5.0 (±3.2) ×10^{-3}	0.26 ± 0.08	0.9977
230	4.6 (±0.3) ×10^{-2}	1			0.9962

Table S8. Observed first-order rate constant and normalized amplitude parameters for the fit of the mono- and biexponential functions to the substrate radical decay kinetics at different temperatures for 100 mM aminoethanol, with 2% v/v added glycerol cryosolvent.

T (K)	$k_{obs,f}$ (s^{-1})	$A_{obs,f}$	$k_{obs,s}$ (s^{-1})	$A_{obs,s}$	R^2
203	1.2 (±0.0) ×10^{-3}	0.42 ± 0.02	3.3(±0.1) ×10^{-4}	0.58 ± 0.02	0.9979
207	1.6 (±0.3) ×10^{-3}	0.52 ± 0.13	5.5 (±1.6) ×10^{-4}	0.48 ± 0.13	0.9960
208	2.2 (±0.2) ×10^{-3}	0.59 ± 0.13	8.7 (±0.8) ×10^{-4}	0.41 ± 0.13	0.9972
210	3.7 (±0.7) ×10^{-3}	0.50 ± 0.25	1.4 (±0.1) ×10^{-3}	0.50 ± 0.25	0.9980
212	4.6 (±1.4) ×10^{-3}	0.60 ± 0.38	1.6 (±0.2) ×10^{-3}	0.40 ± 0.38	0.9968
214	7.4 (±2.4) ×10^{-3}	0.46 ± 0.25	3.2 (±0.1) ×10^{-3}	0.54 ± 0.25	0.9975
217	1.1 (±0.1) ×10^{-2}	0.52 ± 0.22	5.0 (±1.8) ×10^{-3}	0.48 ± 0.22	0.9976
219	1.0 (±0.1) ×10^{-2}	1			0.9967
220	1.3 (±0.0) ×10^{-2}	1			0.9962
223	2.1 (±0.0) ×10^{-2}	1			0.9963

Table S9. Observed first-order rate constant and normalized amplitude parameters for the fit of the mono- and biexponential functions to the substrate radical decay kinetics at different temperatures for 100 mM aminoethanol, with 2% v/v added DMSO cryosolvent.

T (K)	$k_{obs,f}$ (s^{-1})	$A_{obs,f}$	$k_{obs,s}$ (s^{-1})	$A_{obs,s}$	R^2
196	3.3 (±0.7) ×10^{-4}	0.37 ± 0.03	6.3(±3.7) ×10^{-5}	0.63 ± 0.03	0.9954
200	7.2 (±0.3) ×10^{-4}	0.41 ± 0.10	1.6 (±0.2) ×10^{-4}	0.59 ± 0.10	0.9967
202	1.3 (±0.1) ×10^{-3}	0.29 ± 0.03	3.3 (±0.1) ×10^{-4}	0.81 ± 0.03	0.9984
204	2.3 (±0.3) ×10^{-3}	0.22 ± 0.06	5.3 (±0.8) ×10^{-4}	0.78 ± 0.06	0.9987
206	2.8 (±0.4) ×10^{-3}	0.26 ± 0.05	7.6 (±1.1) ×10^{-4}	0.74 ± 0.05	0.9984
208	1.4 (±0.1) ×10^{-3}	1			0.9973
210	2.2(±0.5) ×10^{-3}	1			0.9912
212	3.2 (±0.4) ×10^{-3}	1			0.9961
214	4.9 (±0.4) ×10^{-3}	1			0.9922
216	6.6 (±1.4) ×10^{-3}	1			0.9822
218	9.7 (±2.8) ×10^{-3}	1			0.9714
220	1.3 (±0.4) ×10^{-2}	1			0.9295
222	2.2 (±0.6) ×10^{-2}	1			0.8919
224	2.4 (±0.2) ×10^{-2}	1			0.8687

Table S10. Values of the Arrhenius activation energy (E_a) and prefactor (A; as ln$A$) obtained from linear fits to the decay kinetics of substrate radical states, for the different components (Figure S3). Decay components correspond to the T-dependence of the observed rate constants, $k_{obs,m}$ (mono, $\mathbf{S}^{\bullet}$ decay; T greater than T of order-disorder transition), and $k_{obs,s}$ and $k_{obs,f}$ (slow, $\mathbf{S_1}^{\bullet}$ and fast, $\mathbf{S_2}^{\bullet}$ decays, respectively; T less than T of order-disorder transition). The standard deviation is obtained from the 95% confidence interval for the fit, under the assumption of the normal error distribution.

**Condition**	**Component**	**ln[A (s^{-1})]**	**E_a (kcal/mol)**
all	mono	20.9 ± 3.1	13.5 ± 0.5
ctrl	fast	40.1 ± 2.6	19.1 ± 2.2
	slow	56.2 ± 2.5	26.4 ± 2.1
+sucrose	fast	29.4 ± 4.8	14.8 ± 4.2
	slow	38.5 ± 5.8	19.6 ± 5.1
+glycerol	fast	30.1 ± 3.7	14.9 ± 3.1
	slow	35.7 ± 3.7	17.7 ± 3.1
+dmso	fast	38.4 ± 5.2	18.1 ± 4.1
	slow	43.3 ± 4.3	20.6 ± 3.4

References

(1) Ionescu, A.; Li, W.; Nforneh, B.; Warncke, K. Coupling of ethanolamine ammonia-lyase protein and solvent dynamics characterized by temperature-dependence of the EPR spin probe mobility and dielectric permittivity. *J. Chem. Phys.* **2021**, *154*, 175101. DOI: 10.1063/5.0040341.

(2) Poole, C. P.; Farach, H. A. Saturation methods for determining relaxation times. In *Relaxation in Magnetic Resonance*, Poole, C. P., Farach, H. A. Eds.; Academic Press, 1971; pp 17-29.

(3) Weil, J. A.; Bolton, J. R. *Electron Paramagnetic Resonance: Elementary Theory and Practical Applications*; Wiley, 2007.

(4) Eaton, G. R.; Eaton, S. S.; Barr, D. P.; Weber, R. T. *Quantitative EPR*; Springer Vienna, 2010. DOI: 10.1007/978-3-211-92948-3.

(5) Poole, C. P.; Farach, H. A. The Bloch Equations. In *Relaxation in Magnetic Resonance*, Poole, C. P., Farach, H. A. Eds.; Academic Press, 1971; pp 4-16.

(6) Dalal, D. P.; Eaton, S. S.; Eaton, G. R. The effects of lossy solvents on quantiative EPR studies. *J. Magn. Reson.* **1981**, *44*, 415-428. DOI: 10.1016/0022-2364(81)90276-6.

(7) Kohne, M.; Zhu, C.; Warncke, K. Two dynamical regimes of the substrate radical rearrangement reaction in B12-dependent ethanolamine ammonia-lyase resolve contributions of native protein configurations and collective configurational fluctuations to catalysis. *Biochemistry* **2017**, *56*, 3257-3264. DOI: 10.1021/acs.biochem.7b00294.
